# The global dissemination of hospital clones of *Enterococcus faecium*

**DOI:** 10.1186/s13073-021-00868-0

**Published:** 2021-03-30

**Authors:** Sebastiaan J. van Hal, Rob J. L. Willems, Theodore Gouliouris, Susan A. Ballard, Teresa M. Coque, Anette M. Hammerum, Kristin Hegstad, Hendrik T. Westh, Benjamin P. Howden, Surbhi Malhotra-Kumar, Guido Werner, Katsunori Yanagihara, Ashlee M. Earl, Katherine E. Raven, Jukka Corander, Rory Bowden, Enterococcal Group, Mette Pinholt, Katherine Loens, Basil B. Xavier, Veerle Matheeussen, Herman Goossens

**Affiliations:** 1 Department of Infectious Disesase and Microbiology, Royal Prince Alfred Hospital, Sydney, NSW Australia; 2 University of Sydney, Sydney, NSW Australia; 3 Department of Medical Microbiology, University Medical Center Utrech, Utrecht, The Netherlands; 4 Cambridge University Hospitals NHS Foundation Trust, Cambridge, UK; 5 Microbiological Diagnostic Unit Public Health Laboratory, The University of Melbourne at The Peter Doherty Institute for Infection and Immunity, Melbourne, Victoria Australia; 6 Department of Microbiology, Ramón y Cajal University Hospital and Ramón y Cajal Health Research Institute (IRYCIS), Madrid, Spain; 7 Network Research Centre for Epidemiology and Public Health (CIBERESP), Madrid, Spain; 8 Statens Serum Institut, 2300 Copenhagen S, Denmark; 9 Department of Microbiology and Infection Control, Norwegian National Advisory Unit on Detection of Antimicrobial Resistance, University Hospital of North-Norway, Tromsø, Norway; 10 Research Group for Host-Microbe Interactions, UiT – the Arctic University of Norway, Tromsø, Norway; 11 MRSA Knowledge Center, Department of Clinical Microbiology, Hvidovre Hospital, Hvidovre, Denmark; 12 Laboratory of Medical Microbiology, Vaccine & Infectious Disease Institute, Universiteit Antwerpen, Wilrijk, Belgium; 13 National Reference Centre for Staphylococci and Enterococci, Division of Nosocomial Pathogens and Antibiotic Resistances, Department of Infectious Diseases, Robert Koch Institute, Wernigerode Branch, Wernigerode, Germany; 14 Department of Laboratory Medicine, Nagasaki University Graduate School of Biomedical Sciences, Nagasaki, Japan; 15 Infectious Disease & Microbiome Program, Broad Institute, Cambridge, MA USA; 16 Department of Medicine, University of Cambridge, Cambridge, UK; 17 Department of Biostatistics, University of Oslo, Oslo, Norway; 18 Parasites and Microbes, Wellcome Sanger Institute, Hinxton, Cambridge, UK; 19 The Walter and Eliza Hall Institute of Medical Research, Parkville, Victoria 3052 Australia; 20 Department of Medical Biology, University of Melbourne, 1G Royal Parade, Melbourne, Victoria Australia; 21 Wellcome Centre for Human Genetics, University of Oxford, Roosevelt Drive, Oxford, OX3 7BN UK

## Abstract

**Background:**

The hospital-adapted A1 group of *Enterococcus faecium* remains an organism of significant concern in the context of drug-resistant hospital-associated infections. How this pathogen evolves and disseminates remains poorly understood.

**Methods:**

A large, globally representative collection of short-read genomic data from the hospital-associated A1 group of *Enterococcus faecium* was assembled (*n* = 973). We analysed, using a novel analysis approach, global diversity in terms of both the dynamics of the accessory genome and homologous recombination among conserved genes.

**Results:**

Two main modes of genomic evolution continue to shape *E. faecium*: the acquisition and loss of genes, including antimicrobial resistance genes, through mobile genetic elements including plasmids, and homologous recombination of the core genome. These events lead to new clones emerging at the local level, followed by the erosion of signals of clonality through recombination, and in some identifiable cases producing new clonal clusters. These patterns lead to new, emerging lineages which are able to spread globally over relatively short timeframes.

**Conclusions:**

The ability of A1 *E. faecium* to continually present new combinations of genes for potential selection suggests that controlling this pathogen will remain challenging but establishing a framework for understanding genomic evolution is likely to aid in tracking the threats posed by newly emerging lineages.

**Supplementary Information:**

The online version contains supplementary material available at 10.1186/s13073-021-00868-0.

## Background

*Enterococcus faecium,* a commensal of the gastrointestinal tract, is a common cause of serious hospital-associated infections [1]. Therapy is complicated by resistance to multiple antibiotics, including vancomycin; as a consequence, the World Health Organization includes vancomycin-resistant *E. faecium* (VRE) on its list of priority multidrug-resistant pathogens. *E. faecium* tends to persist in the hospital environment, leading to outbreaks without clear transmission chains and the dissemination of antimicrobial resistance from a variety of sources [2], making the control of hospital *E. faecium* challenging.

Vancomycin resistance occurs in 30–50% of isolates in some countries and is considered the greatest threat to successful treatment. Vancomycin resistance is almost always linked to the presence of the *vanA* and/or *vanB* gene cluster, whose relative frequencies vary in space and time [3, 4]. Optimal therapy for VRE infections remains uncertain; daptomycin and linezolid are the most commonly utilised last-line antibiotics. Besides plasmid-mediated linezolid resistance leading to sporadic outbreaks, resistance to last-line antibiotics remains uncommon [5]. Mutations in any of three genes (*liaF*, *liaS*, *liaR*) linked to cell wall stress responses, or in genes for cardiolipin synthase (*cls*) or glycerophosphoryl diester phosphodiesterase (*gdpD*), may confer daptomycin resistance, while mutations in 23S rRNA, the Cfr rRNA methyltransferase gene or *optrA* may lead to linezolid resistance [6, 7]. It remains unclear whether these resistance mutations arise preferentially on particular gene sequences or are associated with specific circulating clones.

Isolates of *E. faecium* can be placed in two genomically distinguishable groups, also referred to in the literature as “lineages” or “clades”: a hospital-associated lineage (clade A) and a community-associated lineage (clade B) [8]. Clade A is further split into clade A1, represented by human clinical isolates, and several non-A1 sub-clades [9] that, together with clade B isolates, are rarely found in hospitalised patients [10]. Previous genomic studies of A1 *E. faecium* have revealed substantial levels of genome plasticity and evidence of clonal outbreaks in individual hospitals [2]. At the scale of national or regional surveillance, patterns of A1 evolution become more complex; for example, new clone(s) have been reported that have outcompeted and ultimately replaced existing clones (e.g. *vanA* ST1421 in Australia or *vanB* ST192 in Germany) [3, 4]. How local institutional and regional factors, including infection control and antimicrobial stewardship, shape the global population structure of hospital *E. faecium*, and specifically clade A1, remains uncertain.

Standard genomic analyses of bacterial evolution employ phylogenetic modelling of mutations occurring in the core genome, identified either by mapping to a reference or identifying SNPs in core genes in de novo assemblies. Both approaches disregard much of the information that impacts inferences of genomic relationships. In addition, when substantial ongoing recombination is the main driver of genomic diversity, phylogenetic methods fail to accurately capture co-ancestry relationships, i.e., they fail to link genomes that share the most recent ancestors for the largest proportion of their genome (and may instead group together genomes which have independently acquired a similar recombinant sequence) [11, 12]. Accordingly, we adopted a combined analysis approach to examine the population structure and evolutionary dynamics of this important hospital pathogen by using similarity in gene content and proximity in co-ancestry to recover genomic relationships. Our approach attempts to maximise the utilisation of genomic information for a large, globally representative collection of A1 *E. faecium* isolate sequences. It defines genomic relationships based on the presence or absence of genes within the pan-genome, with subsequent fine-tuning based on admixture patterns of core-genome SNPs. These data have allowed us to investigate the size and structure of the Group A1 pan-genome, the population structure that has formed during its worldwide dissemination, and signatures of repeated acquisition of antimicrobial resistance, providing a new description of the mechanisms involved in the ongoing evolution of a globally significant pathogen.

## Methods

### Defining A1 group isolates

Short-read sequence data from a total of 1100 *E. faecium* isolates were contributed by study investigators. Of these, 321 were newly sequenced and uploaded to NCBI under project number PRJNA63689 [13], with the remaining sequences (*n* = 774) downloaded from NCBI. Five isolates failed quality checks (using fastQC v0.11.9) after adapter trimming using trimmomatic v0.38 [14] and were excluded. To enable previous grouping, the dataset was supplemented with 52 [clade A1 (*n* = 14), clade A2 (*n* = 28) and clade B (*n* = 10)] additional sequences from Lebreton et al. [8]. Reads were mapped to Aus0004 (a closed annotated Australian genome, GenBank: CP003351) using bwa [15]. Single nucleotide polymorphisms (SNPs) were identified in each isolate using FreeBayes [16] with alleles filtered for read depth (> 20), and mapping quality, requiring 90% of reads to support a variant allele call. The final SNP matrix included only variant sites present in > 75% of isolates. Using hierBAPS [17] with 2 levels of hierarchy and maxK of 20, isolates were assigned to A1 (*n* = 997), A2 (*n* = 109) and B (*n* = 41) groups [8]. Isolate details and associated metadata can be found in Additional file 1: Table S1.

### Pan-genome analysis and clustering of A1 hospital-associated isolates

Non-enterococcal reads, based on taxonomic k-mer matches using kraken2 [18], were discarded to prevent subsequent spurious gene calls as a result of contamination. Sequences were assembled using SPAdes v3.13.1 [19] under the “careful” option, with assemblies failing quality metrics excluded (*n* = 24). Contigs < 2000 bp were removed and discarded from individual assemblies, which were annotated using prokka v1.13 [20] prior to pan-genome discovery using panaroo v1.1.2 [21] under the “sensitive” mode. This setting is optimised to find all genes present in all isolates with homologous genes classified when gene sequences differ by > 5% between isolates. The identified panaroo core-genome was larger than previous estimates and therefore the analysis was duplicated using Roary [22].

The panaroo output, a gene presence-absence matrix, was used to cluster isolates after initial dimension reduction using Barnes-Hut t-Distributed Stochastic Neighbour Embedding in R with the Rtsne v0.15 package https://github.com/jkrijthe/Rtsne [23]. The optimal number of clusters was evaluated with aid of the fviz_nbclust function within the R factoextra v1.0.6 package https://github.com/kassambara/factoextra [24].

Subsequently, cluster membership was refined using the ChromoPainter tool embedded in FineSTRUCTURE v2.1.3 [25] with a starting mean recombination rate of 2.3 × 10^−7^. This method models each genome as a succession of haplotype fragments from other genomes in the collection and assigns the closest matching sequence as the donor haplotype with switches between donors occurring at ancestral recombination breakpoints. All these genome sections can then be ascribed or “painted” according to their inferred origins among groups of related donor sequences. Isolates with similar admixture patterns across the majority of their genome, representing a continuous line of ancestry, signify specific lineages. These lineages coalesce to form clusters with isolates expected to share the greatest proportion (always > 35%) of their co-ancestry with another isolate assigned to the same cluster. Conversely, highly admixed isolates, as a result of numerous recombination events, could not be assigned to any pan-genome assigned cluster and were excluded from further analysis. For admixed regions, the likely “donor” isolate (and by inference the country of origin) can be found by looking for the isolate that contains the most similar sequence in the alternative clusters.

Final refined clustering of A1 isolates can be found in Additional file 1: Table S1.

### Phylogenetic and spatial analysis

Maximum clade credibility trees were constructed on cluster-specific SNP matrices, excluding isolates with unknown collection dates, following masking of identified recombination sites using ClonalFrameML [26]. For each relevant cluster, several population models (constant, exponential and skyline) in combination with various clock models (strict, relaxed exponential and random) were run for 3 × 10^8^ iterations using BEAST v2.6.1 [27]. Resulting trees were pruned, and a maximum clade credibility tree was obtained from the optimally performing model.

A spatial analysis was performed for Cluster 2, with inclusion of a geographical trait based on latitude and longitude co-ordinates and phylogenetic nodes assigned to a geographical location based on ancestral state reconstruction. Transmission links for isolates and nodes were inferred using genomic, spatial and temporal variables and visualised using SpreaD3 [28] under a discrete trait model. All isolates and nodes are represented by a network of connecting lines depicting the likely “origin” and “destination”. Lines are coloured according to “destination” location.

### MLST, resistome, virulome and plasmidome analysis

Multi-locus sequence typing was performed using the de novo assemblies. Isolate resistomes were predicted using AMRfinder specifying *E. faecium* [29]. Mutation-based resistance for daptomycin was predicted by annotation of variants against the Aus0004 genome. A median-joining network of the *liaFSR* genes was constructed using NETWORK v10.0 [30]. 23S rRNA-based linezolid resistance was predicted as previously described [31]. The virulome was predicted using blastn against the downloaded virulence factor database [32]. To determine genes likely associated with plasmids, we implemented mlplasmids [33] using de novo assemblies with a posterior probability cut-off of 0.7.

### Statistical analysis

Statistical analyses for associations were performed using the appropriate two-sided statistical test following consideration of the data distribution. A *p* value < 0.05 was considered significant, with all calculations performed using the R stats package v 3.4.4 [34].

## Results

### Defining group A1 isolates in a global collection of hospital *E. faecium*

Groups that had previously published *E. faecium* genome data were invited to contribute. Participants were requested to provide short-read sequence data representing randomly selected isolates, spread across time, from collections of vancomycin-susceptible and vancomycin-resistant *E. faecium* originating from hospitalised patients in any country, ensuring that isolates from the same hospital were not enriched for known outbreaks, based on local epidemiological data.

Sequence variation identified through mapping reads to a reference genome was used to cluster isolates with hierBAPS [17], recovering the previously published A1 (*n* = 997), A2 (*n* = 109) and B (*n* = 41) groups. Of the A1 isolates, 24 subsequently failed de novo assembly quality filters and were excluded, leaving 973 A1 isolates originating from 31 countries, spanning a 30-year period (1986–2016) for analysis in this study.

### Heterogeneity among group A1 hospital *E. faecium* isolates

**Fig. 1:**
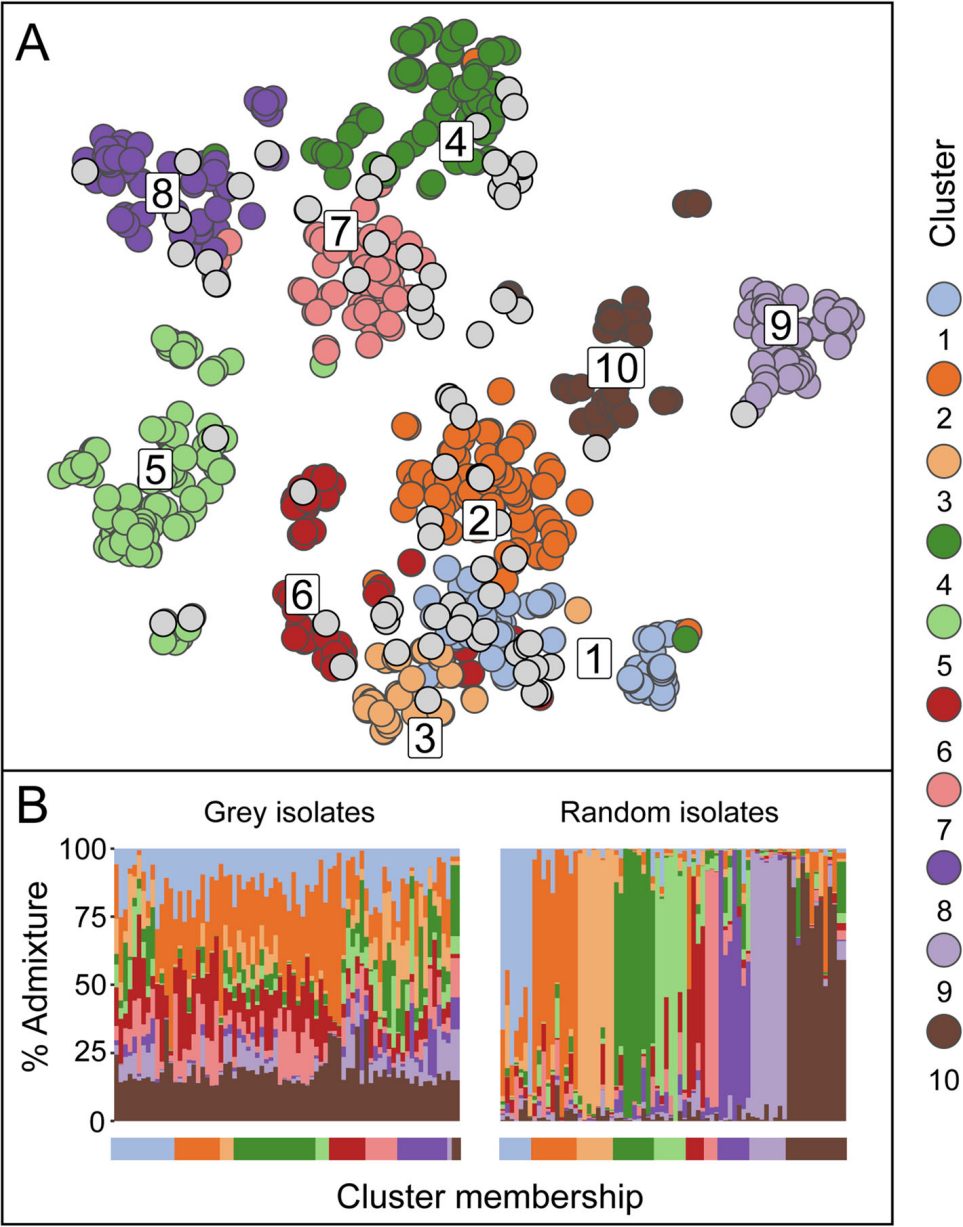
Clustering among global A1 isolates. **a** Clustering of 973 *E. faecium* group A1 isolates based on the presence or absence of genes within the pan-genome identified using Panaroo. Labelled clusters are represented on a reduced dimensionality 2-D grid with member isolates coloured as shown in the legend. At a core-genome SNP level isolates within the same cluster are expected to share the same ancestry across the majority of their genomes. Using ChromoPainter, substantial core-genome admixture was detected in the 78 grey-shaded isolates, resulting in their exclusion from the designated pan-genome clusters. **b** The levels of admixture of the aforementioned 78 grey-shaded isolates in **a** (left) are contrasted with estimated admixture of 78 randomly chosen isolates from the remaining 895 isolates. The *x*-axis label shows the initial cluster assignment based on the pan-genome with the *y*-axis bars representing co-ancestry signals originating from other clusters, using the same colours as in **a**

To study the relationships among *E. faecium* A1 isolates, a pan-genome analysis based on de novo-assembled genomes, investigating large-scale variation comprising the presence and absence of genes and distinct forms of genes, was undertaken using Panaroo [21]. The 973 A1 isolates were separated into 10 clusters (Fig. 1a).

Recognising the role of homologous recombination as a dominant, recurring force in the continuing genomic evolution of *E. faecium*, the assignment of isolates to pan-genome clusters was refined using chromosome painting [25]. This analysis traces the signal of co-ancestry shared between isolates, based on reads mapped to a reference core genome sequence of 1926 genes (those present in > 95% of isolates), and provides a “fingerprint” that distinguishes all but the most closely related isolates. Chromosome painting identified 78 isolates, typically visualised at the edges of the pan-genome clusters (Fig. 1a, b), whose core genomes revealed substantial ancestry in multiple clusters. For the remaining 895 isolates, aggregating fragments of sequence including those transferred between lineages, a mean of 81% (IQR 75–89%) of an isolate’s core genome shared co-ancestry with other isolates within the cluster.

### Pan-genome and plasmidome

**Fig. 2:**
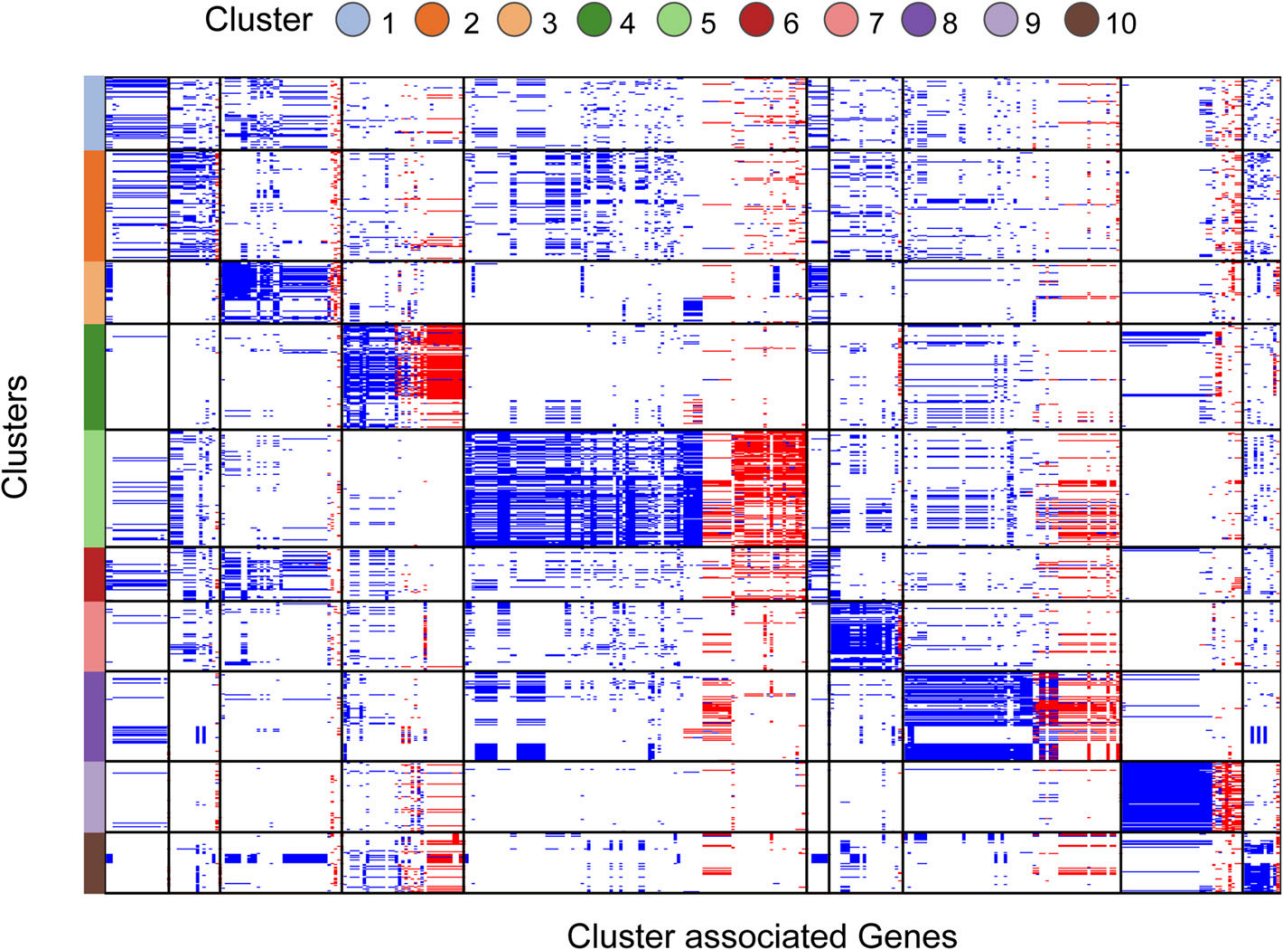
Cluster-associated genes. All 366 genes significantly associated (*p* < 0.05) with pan-genome clusters are depicted with chromosomal and plasmid-derived genes coloured blue and red respectively. Of the 366 cluster-associated genes, 129 genes occurred on a plasmid in at least one isolate. Genes (along the *x*-axis) are grouped by cluster (along the *y*-axis) as depicted in the legend. No gene was exclusively limited to any one cluster, with the largest complement of genes associated with cluster 5 (*n* = 107)

Panaroo identified 15,788 genes and gene variants among A1 isolates, with a median genome size of 2781 identifiable genes. Along with 1926 genes present in > 95% of isolates, 2084 “shell” genes were found in 10–95% of genomes, arranged in groups of similar frequency hinting at assemblages of genes shared across multiple clusters. These estimates were recapitulated using Roary [22] and are larger than previously reported, indicating the influence of non-human isolates in limiting core-genome size estimates in other studies [10]. A substantial proportion (854; 40.9%) of genes were identified on plasmids (Additional file 2: Figure S1), providing evidence of plasmid gain contributing to cluster emergence and diversification with 129 of the 366 cluster-associated genes occurring on a plasmid in at least one isolate (Fig. 2). The total frequencies of plasmid-derived genes were similar across clusters. Most of the remaining 11,778 genes (76% of the total) were found in no more than a handful of closely related isolates (< 5% of isolates), reflecting in each case a probable single acquisition from an outside source. Although the average nucleotide diversity for core genes was low (pi = 0.0056); substantial variability, through the identification of homologues with > 5% divergence, in some ubiquitous genes including housekeeping genes (*adk, atpA* and *pstS*) was noted. These patterns likely indicate a single clonal expansion leading to A1 with infrequent replacement of functionally constrained genes from outside the *E. faecium* species.

### Patterns of A1 dissemination and admixture

Genome-wide nucleotide diversity among A1 *E. faecium* isolates is small, stemming from its origin as a hospital-restricted clone. Nevertheless, it is possible to detect core genome lineages that have arisen by a combination of mutation and admixture with non-A1 *E. faecium*. Of the 10 clusters, some (9, 10) were found almost entirely in one geographic location (country), while others were limited to geographical regions (clusters 3 and 4 in Asia and clusters 5 and 7 in Europe). Samples in the remaining clusters (1, 2, 6, 8) originated from highly overlapping, indistinct territories within Europe or internationally (Additional file 2: Figure S2). The genome sequences assigned to each of the generalised European/world clusters typically include many of the oldest samples (especially cluster 2) in contrast with geographically specific clusters (Additional file 2: Figure S3). The relationship between time-depth of sampling and diversity suggests that A1 has evolved and disseminated over timescales comparable with sampling times, i.e. decades.

**Fig. 3:**
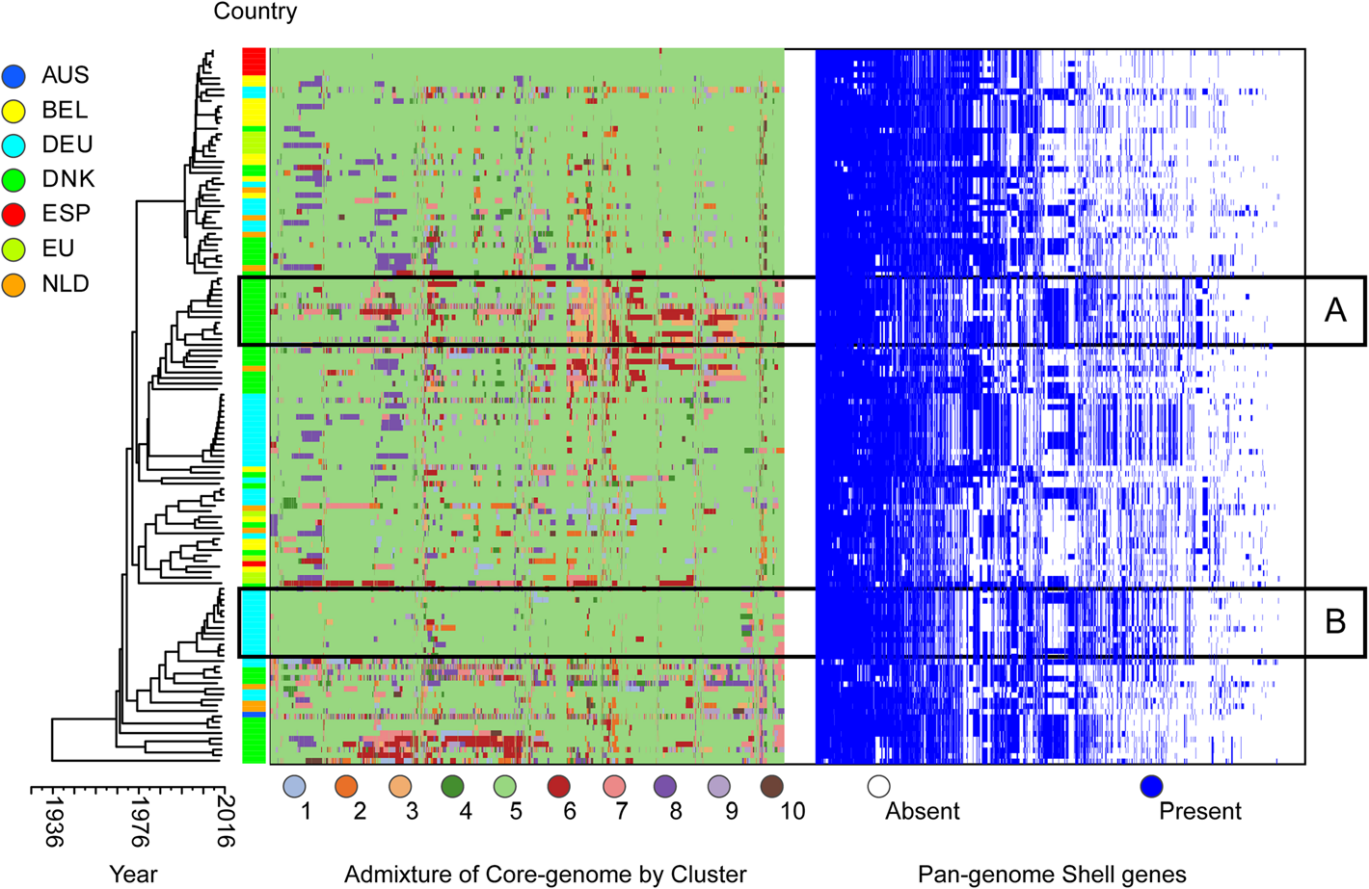
Cluster 5: Evidence of local adaptation and regional dissemination. Maximum clade credibility tree of 129 cluster 5 isolates following masking of recombination. The first column to the right of the tree is coloured by sampled country (key left upper corner: AUS, Australia; BEL, Belgium; DEU, Germany; DNK, Denmark; ESP, Spain; EU, European Union; NLD, Netherlands). The subsequent column or heatmap depicts admixture events across the core genes of isolates coloured by “donating” cluster (key below heatmap). Similar background patterns of admixture are observed, accompanied by evidence of ongoing recombination at regional levels. Two such instances linked to Denmark and Germany are highlighted by boxes A and B respectively. Time scale is shown on the *y*-axis below the phylogeny

We next focus on three selected clusters to examine and illustrate distinct patterns of adaptation, admixture between clusters and networks of spread. Cluster 5 emerged in Denmark before disseminating across Northern Europe (Fig. 3). The single Australian isolate within cluster 5 is suggestive of wider spread; however, the identified expansion seems predominantly restricted to mainland Europe. Substantial recombination (involving all core genes) has resulted in highly variable patterns of admixture at the gene level. However, isolates from a single country retain a strong recent clonal signature inclusive of the pan-genome (depicted in panels A and B in Fig. 3), superimposed on a deeper structure of emergence, adaptation and expansion prior to spread. The scatter of Dutch isolates, for example, across the cluster phylogeny, reveals a pattern of homogenisation across Europe, leading to a picture of the co-circulation of multiple subclones that could not previously be appreciated; these co-circulating lineages augment the pool of core-genome variants and accessory genes that can contribute to the emergence of new lineages (Fig. 3).

**Fig. 4:**
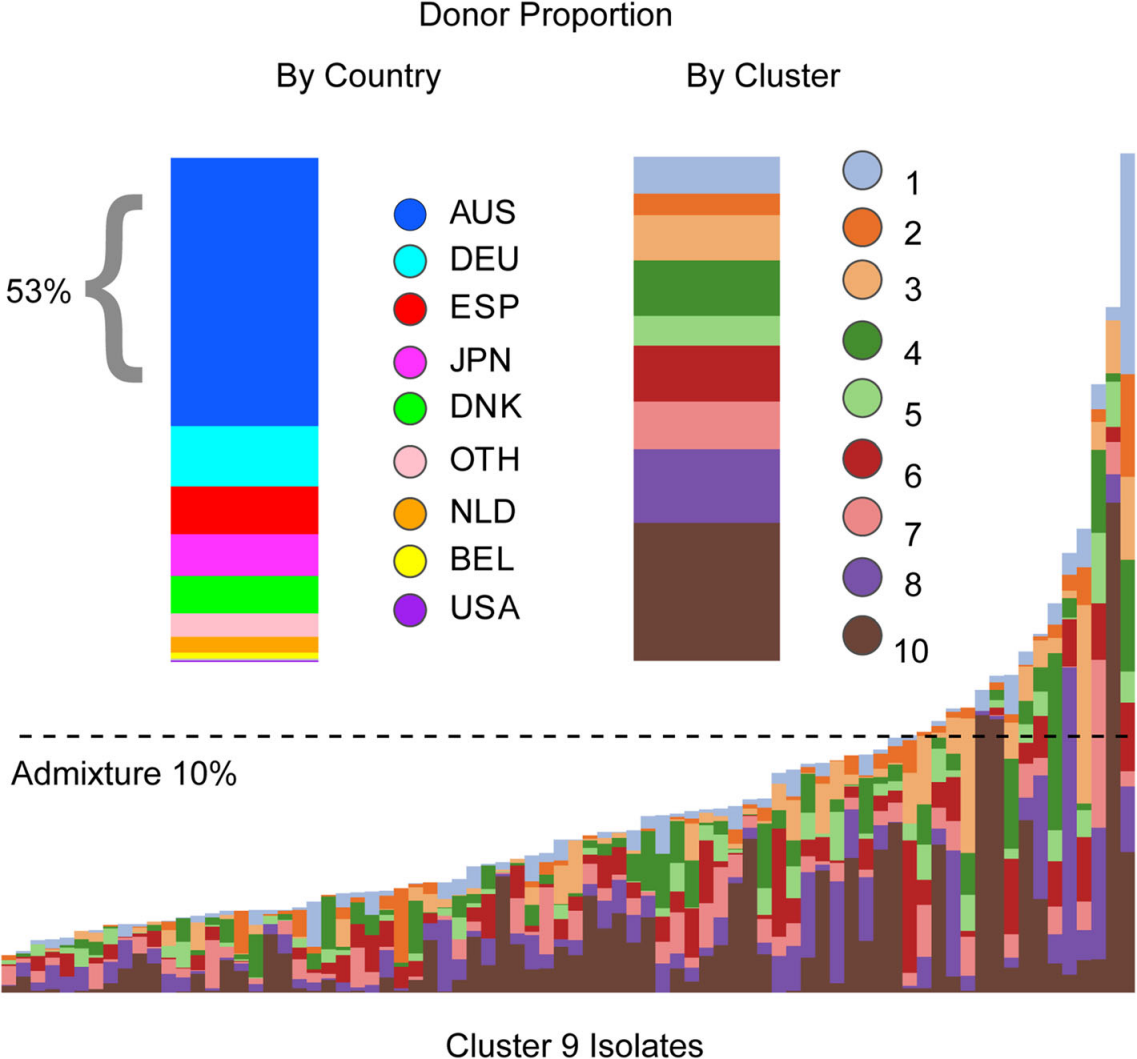
Cluster 9. The bottom panel along the *x*-axis represents individual cluster 9 isolates (originating exclusively from Australia) with coloured vertical bars showing individual admixture signals across their core-genome by donor cluster. The dashed line depicts an admixture threshold of 10%. The top half of the panel reflects the proportions of admixed regions by donor country (left) and donor cluster (right). Overall, cluster 9 isolates share the majority of their genome co-ancestry (between 61% and 98%) with other cluster 9 isolates. In admixed regions, the best genomic match to the donor cluster originated from another Australian isolate in 53% of cases

To appreciate the granularity of the patterns of admixture and genetic exchange between clusters and isolates, focus is switched to cluster 9, consisting exclusively of isolates from Australia. The collection of cluster 9 isolates in multiple institutions in several cities rules out a single outbreak or clonal expansion event as a simple explanation for this cluster (data not shown). Signals of recombination are observed across all genes, incorporating sequences characteristic of all other clusters, in spite of the low frequencies of some clusters in Australia. In fact, local isolates provided the best match for a large proportion (53%) of copying events from other clusters (Fig. 4), confirming the duality of local acquisition of sequences and links to the worldwide store of core genome variation. Sampling dates for cluster 9 were closely spaced, obscuring the relationship between time and diversification while remaining consistent with a rapid emergence.

**Fig. 5:**
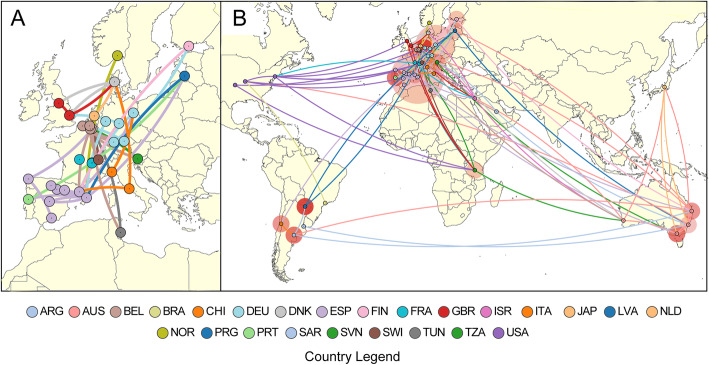
Dissemination routes of *E. faecium* across the world. Worldwide dissemination of genomes belonging to cluster 2 (*n* = 122) analysed using spatial, temporal and genomic data through BEAST v2.6 and visualised using SpreaD3. Coloured nodes on the map represent an isolate’s country of origin (as shown in the legend), while connecting lines are coloured by destination location. An expanded view is shown in panel **a** to show inferred spread of *E. faecium* isolates across Europe with complex links between and within countries. **b** *E. faecium* dissemination across the globe with surrounding circle sizes proportional to the number of lineages (isolates that share the same continuous line of ancestry) that occur in that location and captures the absolute and relative intensity of the local spread at any given point in time

**Fig. 6:**
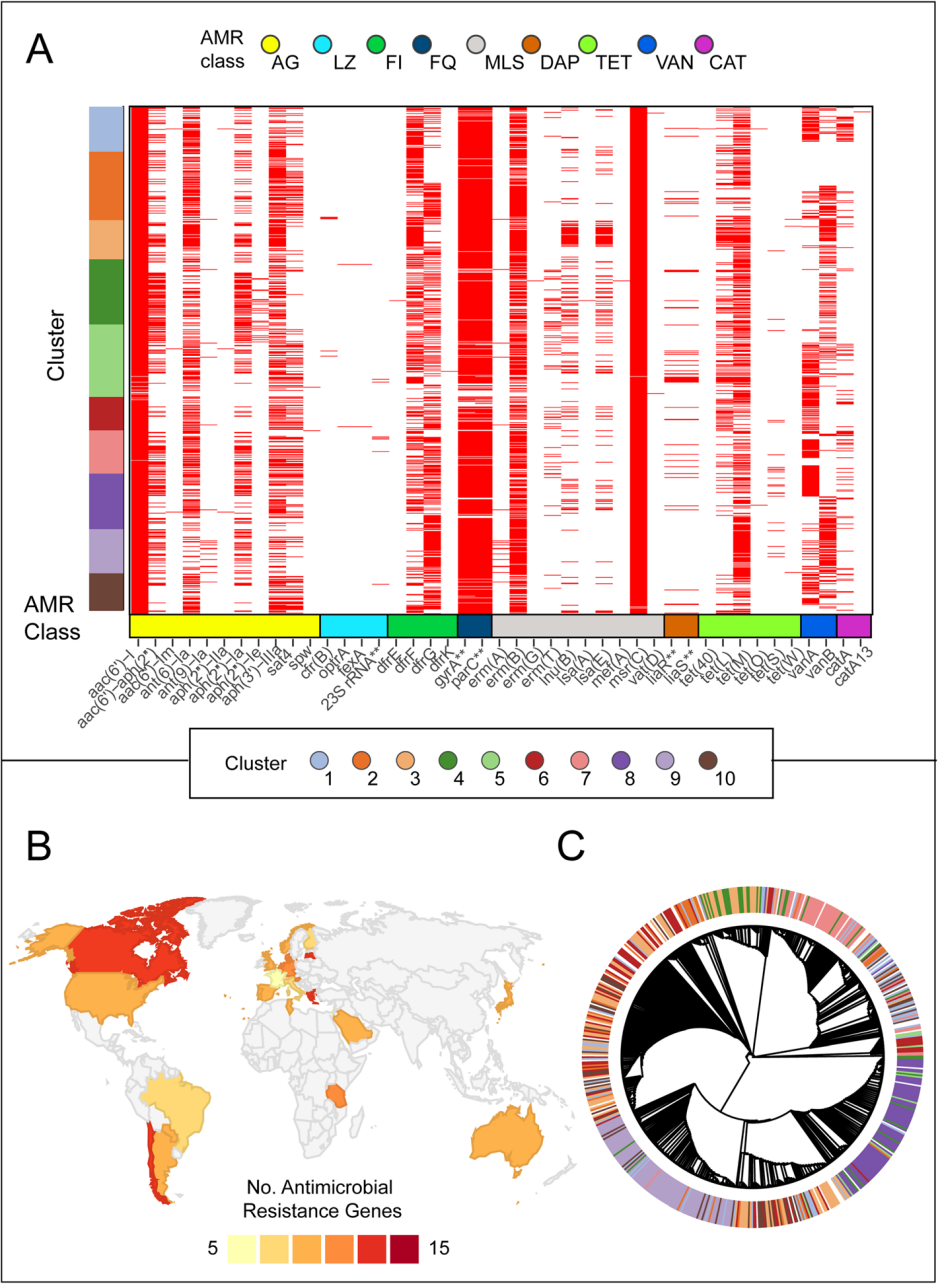
Antimicrobial Resistance. **a** Presence (red) or absence (white) of resistance genes grouped by isolates within Panaroo clusters along the *y*-axis. Genes are named along the *x*-axis with mutational resistance represented by gene names highlighted by 2 asterisks and grouped by AMR class. AG, aminoglycosides; LZ, linezolid; FI, folate inhibitors; FQ, fluoroquinolones; MLS, macrolide-lincosamide-streptogramin; DAP, daptomycin; TET, tetracycline; VAN, vancomycin; and CAT, chloramphenicol. **b** Heatmap of the average number of antimicrobial resistance genes resulting in resistance within isolates by country. No isolates were included from grey shades countries. **c** Circular dendrogram of the *tetM* gene with designated cluster by colour depicted in the outer ring. Evidence of clustering is still seen (e.g. clusters 7, 8 and 9) but extensive transfer of alleles between clusters by horizontal gene transfer is evident

Cluster 2, present in all sampled continents, was chosen to examine the characteristics and routes of global dissemination (Fig. 5). A phylogenetic analysis after masking recombination revealed a similar substructure as in cluster 5, in which clonal patterns are limited by location and blurred by admixture patterns (Additional file 2: Figure S3). Given the considerable spread and time depth of European isolates, the phylogeny is most consistent with introduction(s) of *E. faecium* into the USA from Europe, most probably from the UK. The lack of a consistent pattern of vancomycin resistance genes among most closely linked isolates in different countries suggests the acquisition of vancomycin resistance at a local level (Fig. 6) [9]. Cluster 2’s epicentre remains in Europe, with regional links between Japan and Australia, and North and South America.

### Antimicrobial resistance and virulome

The availability of a globally representative collection of *E. faecium* A1 isolates facilitated a survey of genes associated with antimicrobial resistance (AMR). A vancomycin resistance gene cluster was present in 544 (56%; 261 *vanA*, 283 *vanB*) A1 isolates. This study recapitulated known associations between country and mode of vancomycin resistance. Cluster 9 (predominantly Australian) and cluster 7 (predominantly German) were skewed towards *vanB*, while cluster 6 (European countries) was significantly more likely to harbour *vanA* (*p* < 0.05) (Fig. 6a). The cluster 9 correlation is driven by the dominance of a single multi-locus sequence type, ST796 in that cluster (Additional file 1: Table S1), whereas in clusters 6 and 7 multiple acquisitions of vancomycin resistance are implicated. Thus, the association of *van* type with geography may be in part driven by the short-term success of particular lineages of *E. faecium*. Trimethoprim resistance, encoded by the *dfrG* gene, was similarly clonally associated (data not shown). Less clonal and more dynamic patterns were observed for *tetM* (tetracycline resistance) and aminoglycoside resistance genes, with evidence for loss events and acquisition of multiple distinct gene variants within a cluster.

Several non-synonymous variants in the *liaSFR* gene cluster, including the daptomycin resistance mutations, *LiaR* ^T73C^ and *LiaS*^T120A^ (CARD database) [35], were observed in our collection. Resistance mutations were carried together and emerged from only one of the two common allele backgrounds (Additional file 2: Figure S4), which were positively associated with clusters 1, 3 and 6 (*p* < 0.05). This background was detected in isolates from most sampled countries studied, implying the emergence of daptomycin resistance in most locations, subject to local selection pressure. Although daptomycin is generally used when treating a VRE infection, daptomycin resistance was not associated with vancomycin resistance in our sample set.

The G2576T mutation in 23S rRNA associated with linezolid resistance was detected in 7 isolates, but, unlike daptomycin, no association with clusters could be detected. Plasmid-mediated linezolid resistance was found in two isolates harbouring the *optrA* gene in conjunction with *fexA* (florfenicol-chloramphenicol transporter gene) and in 6 isolates containing the cfr(B) gene.

A1 *E. faecium* isolates carry a median 10 (range 5–15) resistance genes for which country- and region-specific (notably European) frequencies are observed (Fig. 6b). Overall, *E. faecium* tends to be multi-resistant, harbouring quinolone mutations (in both *parC* and gyrA genes) and at least one gene conferring resistance to aminoglycoside, macrolide-lincosamide-streptogramin, tetracycline and antifolate antimicrobials. Over the study period, hospital-associated A1 *E. faecium* has become more resistant, from a median complement of 4 genes in 1986 to 11 in 2016 (Additional file 2: Figure S5). At the gene level, there is evidence of extensive horizontal gene transfer, in the form of multiple clonal backgrounds within and shared between pan-genome clusters. One such example, the *tetM* gene, depicted in Fig. 6c, illustrates a continuum from minimal admixture and ongoing cluster specificity (clusters 7, 8 and 9) to frequent exchanges resulting in loss of cluster signals.

Ten putative virulence genes were detected in the A1 *E. faecium* isolates. Of these, 6 were found in > 75% of isolates and included genes associated with biofilm formation (*bopD*), adhesion (*sgrA*), bile salt hydrolase (*bsh*), collagen binding (*acm*, *clpP*) and metal transporter (*psaA*) genes (Additional file 2: Figure S6). The capsular polysaccharide gene (*cpsF*) was present in less than 1% of isolates and is generally not associated with virulence in *E. faecium*. Differences in virulomes by cluster were typically characterised by the reduced proportion of isolates carrying specific virulence genes. The functional consequences of these differences are unknown. Similarly, whether these observations represent gene loss or gain events is unknown as no specific patterns were observed in relation to time, geography or country of origin.

## Discussion

The A1 clade of *E. faecium* has a worldwide distribution in which it dominates the hospital population of disease-causing *E. faecium* in each location where it has been studied. The low levels of nucleotide diversity among genes common to all isolates are consistent with the emergence of A1 from the expansion of a single clone, with substantial continuing evolution, during the past several decades. The two main modes of genomic evolution that continue to shape *E. faecium* A1’s diversity and population structure are the acquisition and loss of assemblages of genes that form the mobilizable genome (through the acquisition of mobile genetic elements including plasmids), and homologous recombination of the core genome, in which clones that have emerged since A1’s formation are recombined, eroding initial signals of clonality and in some identifiable cases producing new clonal clusters. Both modes occur at large scales and with considerable tempo, complicating large-scale, genome-wide analyses of population history and structure.

In this study of the largest available collection of diverse and globally distributed *E. faecium* genome sequences from hospitalised patients, comprising 973 A1 isolates from 31 countries collected over 30 years, we define a substantial pan-genome. The core genome contained nearly 2000 genes, accompanied by 854 common accessory genes encoded on plasmids (the “shell genome”), plus a much larger number of sporadically captured genes found in no more than a few isolates. Several gene patterns appear to have been acquired independently more than once, leading to geographically specific clusters and providing a strong indication of ongoing interactions among A1 Enterococci in the gastrointestinal tracts of patients, where many of the same genes are recycled into different clusters. Sporadic acquisitions indicate that hospital *E. faecium* also retains access to a much larger pool of potential genes that have failed to become established in successful clusters.

Within each pan-genome cluster, we were able to identify one or more dominant lineages defined as common haplotypes on which are superimposed imported sequences, acquired through homologous recombination, whose origins are in other clusters. These isolates include ones whose core genomes place them mid-way between established lineages (e.g. the grey-coloured isolates depicted in Fig. 1) and provide the potential starting point for new clusters. These patterns reveal a continuing process of formation of clones, over relatively short periods (decades), comprising newly recombined genomes, some of which are then locally or regionally successful while continuing to recombine and gain and lose genes.

A recent analysis of a globally representative sample set estimated the emergence of the hospital-associated A1 *E. faecium* at ~ 302 years ago [36], in marked contrast with estimates as recent as 74 years [8]. This discrepancy is difficult to resolve using existing methods, since accurate phylogenomic analyses rely on the assumptions that recombination occurs across a restricted part of the genome and that these regions can be reliably detected and removed. Our analysis reveals recombination across the whole genome, even in relatively small sample subsets. Consequently, molecular clock approaches are prone to inaccuracies, providing an explanation for wide discrepancies in estimates. Leaving aside phylogenetic analyses, the very low diversity of large parts of the genome and the global distribution of A1 genomes is consistent with the emergence of *E. faecium* A1 over recent decades, followed by global dissemination.

In contrast with a lack of precise information about its date of emergence, our analysis yields some observations relevant to *E. faecium* A1’s origins. The A1 population in Europe contains more distinct lineages (clusters) than in other sampled locations, and this is reflected in the evidently complex history of recombination in cluster 2. In contrast, other well-sampled locations such as Australia reveal a less complex, more geographically specific pattern of sequence exchange. These observations are consistent with Europe as the birthplace of and longest established home of A1, a conclusion that needs to be tested, given the limited number of American isolates in our study and the potential resulting sampling bias.

Our improved understanding of the processes through which A1 *E. faecium* continues to evolve may help us to better understand the dynamics of selectable traits such as antimicrobial resistance. Our analysis suggests that gain and loss of *van* elements (either the *vanA* or the *vanB* cassette) leading to vancomycin resistance is common enough that geographic specificities are an accident of history, driven by chance through the acquisition and spread of a particular cassette in a particular population. Rather than reflecting a genetic propensity to carry a particular *van* type, common *van* loss events are likely to be followed by re-acquisition of the same *van* type simply because it happens to be frequent in that particular population [2, 37]. This pattern (that gene acquisition is most likely to reflect the distribution of circulating genes) is mirrored by the accessory genome as a whole and explains how a particular complement of shell genes (a pan-genome cluster) exists without being completely linked to a set of core genome lineages. The implication of these observations is that the raw materials for phenotypic plasticity may be population-specific in the short term, but subject to global dissemination and mixing over longer timescales.

Our study also reveals the distinct dynamics underlying resistance to the last-line antibiotic, daptomycin. The variants responsible for daptomycin resistance only appear on one of two ancestral forms of the *liaS* gene that are associated with clusters and geography but nevertheless appear in all populations, implying that mutational selection for resistance is only possible in some infections, but the requisite genotypic background may eventually spread by recombination combined with selection.

## Conclusions

The patterns of variation that we describe for A1, the main hospital-adapted lineage of *E. faecium*, illustrate the ease with which new phenotypes are likely to continue to emerge, driven by local variations in selection and access to distinct gene pools via both homologous recombination and an extensively mobilizable pan-genome. These genomic features suggest that controlling the hospital spread of *E. faecium* will remain challenging.

## Supplementary Information

**Additional file 1: Table S1.** Details of *E. faecium* short read sequence data used in the analysis.

**Additional file 2: Figure S1.** Plasmid and Chromosomal associated shell genes**. Figure S2.** Distribution of clusters by country and collection dates**. Figure S3.** Local adaptation and regional dissemination of *E. faecium***. Figure S4.** LiaFSR haplotypes**. Figure S5.** Antibiotic resistance gene accumulation over time**. Figure S6.** Virulome.

## Data Availability

All new short read sequence data (*n* = 321) was uploaded to NCBI under project number PRJNA63689, https://www.ncbi.nlm.nih.gov/sra/PRJNA636894 [13]. The remaining (*n* = 826) sequences were downloaded from NCBI with project numbers and associated metadata provided in Additional file 1: Table S1.
