# Supplementary material for: The global dissemination of hospital clones of Enterococcus faecium
Source: Genome Med. 2021 Mar 30;13:52. doi: 10.1186/s13073-021-00868-0 (PMC8008517; doi:10.1186/s13073-021-00868-0)
Supplement: Supplementary file 2 — Additional file 2: Figure S1. Plasmid and Chromosomal associated shell genes. Figure S2. Distribution of clusters by country and collection dates. Figure S3. Local adaptation and regional dissemination of E. faecium. Figure S4. LiaFSR haplotypes. Figure S5. Antibiotic resistance gene accumulation over time. Figure S6. Virulome. [file 13073_2021_868_MOESM2_ESM.pdf]

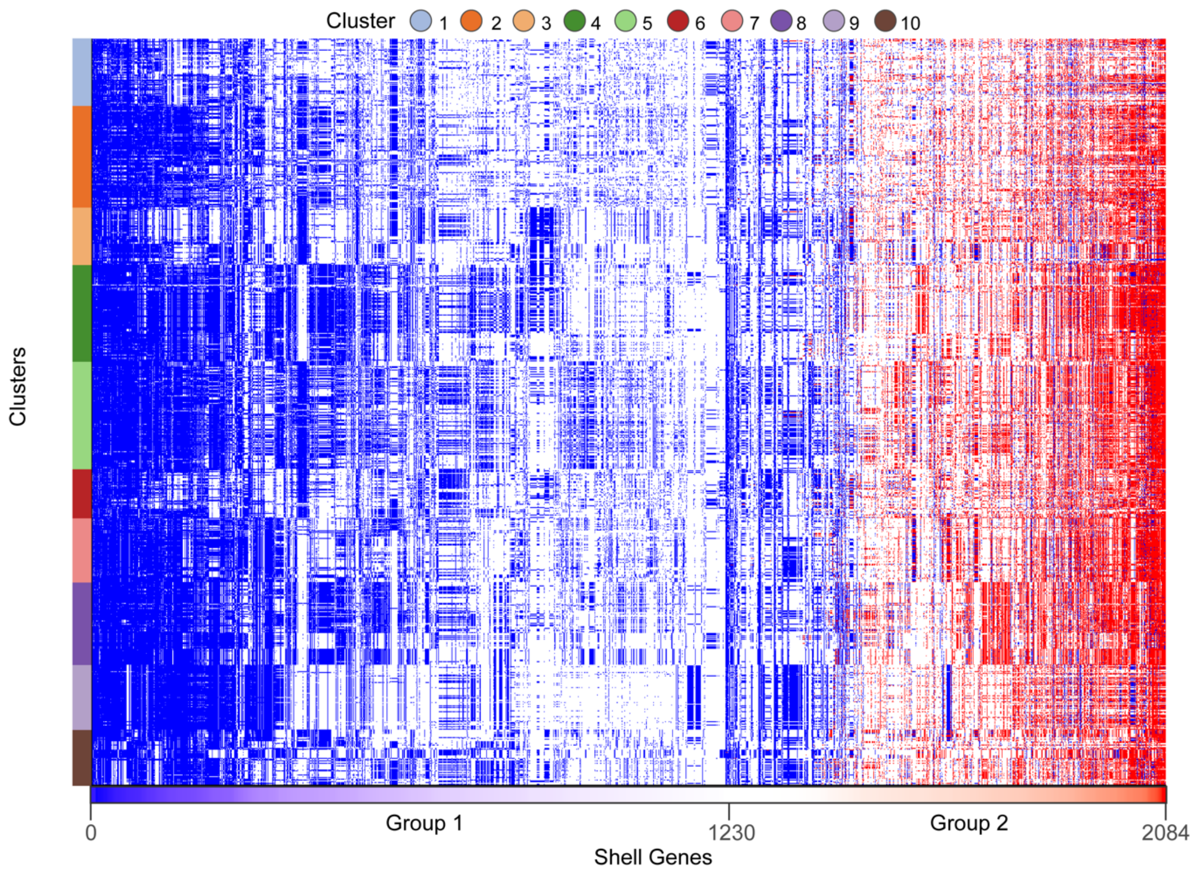

Fig. S1

Plasmid and Chromosomal associated Shell genes.

Shell genes (present in less than 95% but more than 10% of isolates) are depicted for 895 *E. faecium* Group A1 isolates, arranged by cluster (along the y-axis) with chromosomal and plasmid-derived genes present in any isolate-coloured blue and red respectively. Genes are split into two groups (along the x-axis). The first group, ordered by the proportion of isolates containing that gene, represents 1230 genes exclusively contained on the chromosome in all isolates. The second group represent 854 plasmid-derived genes in at least one isolate and is ordered by the proportion of genes located on the chromosome. A frequency plot is depicted below the x-axis.

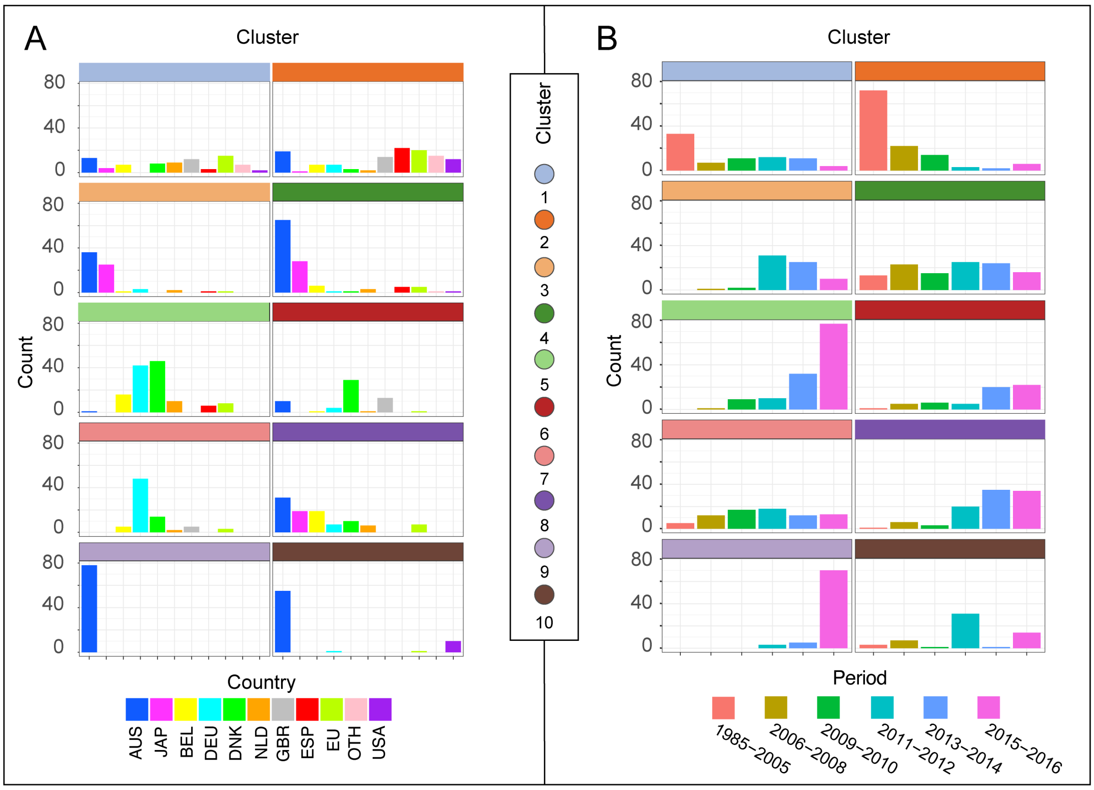

Fig. S2

Distribution of Clusters by Country and by Collection dates.

Distribution of 895 A1 isolates by country of origin (Panel A) and date of sampling (Panel B) grouped by final cluster designation. Countries are grouped by regions Asia (AUS: Australia; JPN: Japan), Europe (BEL: Belgium; DEU: Germany; DNK: Denmark; NLD: Netherlands; GBR: Great Britain; ESP: Spain and EU: Other European Countries); OTH: Various Other countries and USA: United States of America.

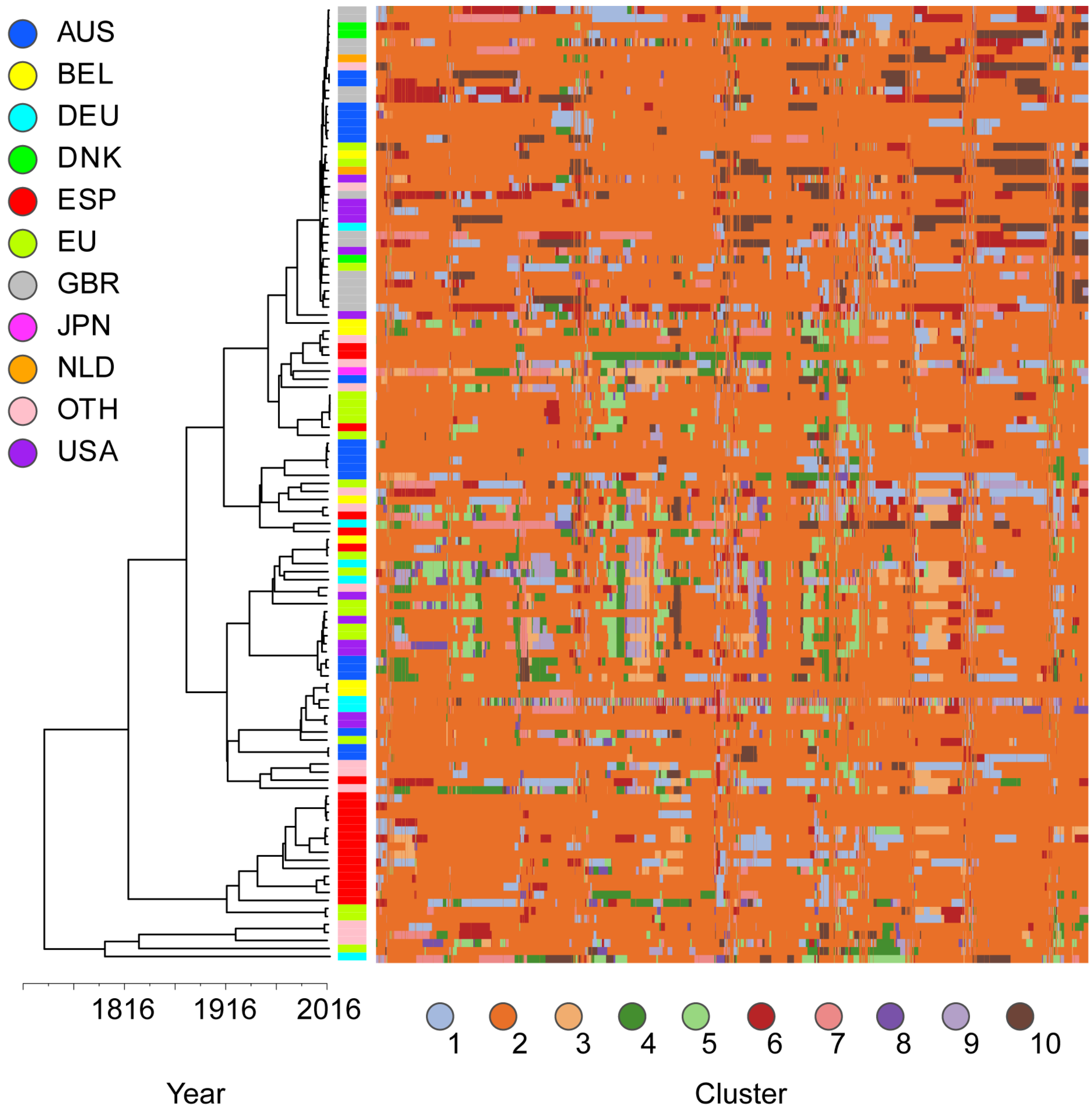

Fig. S3

Local adaptation and regional dissemination of *E. faecium*.

Maximum Clade Credibility Tree of 122 Cluster 2 isolates following masking of recombination. The first column to the right of the tree is coloured by sampled country (key left upper corner: AUS: Australia; BEL: Belgium; DEU: Germany; DNK: Denmark; ESP: Spain; EU: European Union; NLD: Netherlands). The subsequent column or heatmap depicts admixture events across the core genes of isolates coloured by “donating” cluster (key below heatmap).

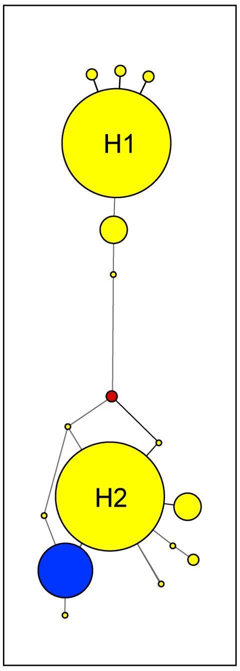

Fig. S4

***liaSFR* Haplotypes.**

Neighbour-joining Network of *liaF, liaS, liaR* non-synonomous mutations depicting 16 encountered haplotypes across the A1 isolates. Daptomycin resistance genotypes LiaR ^{T73C} & LiaS^{T120A} (CARD database) is shown by blue circle and is most likely to have arisen from H2 haplotype. Circle sizes reflect relative frequencies of the haplotype within the population with the red circle depicting a required but absent mutation (within the dataset).

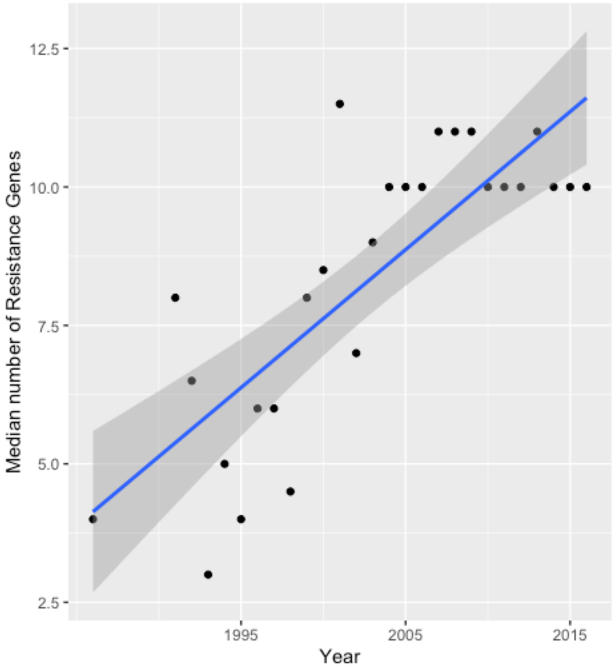

Fig. S5
Antibiotic resistance accumulation over time.
Scatterplot of the median number of antimicrobial resistance genes present in *E. faecium* for year of isolation. Line of best fit and 95% confidence intervals is shown.

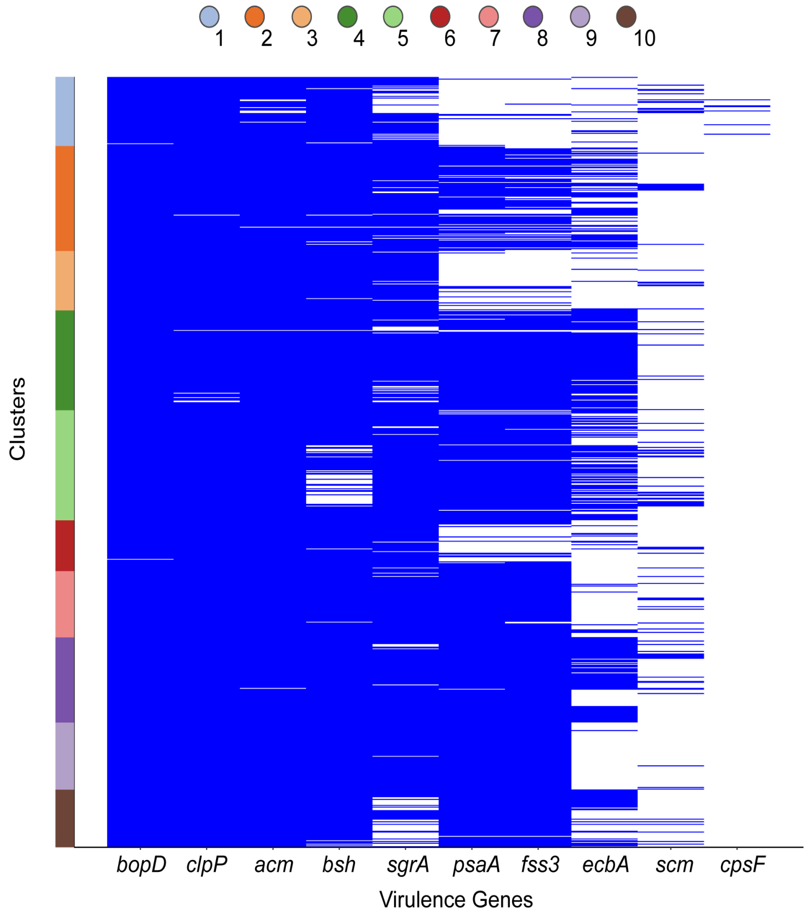

Fig. S6

Virulome.

Presence (blue) and absence (white) of virulence genes grouped by isolate cluster (y-axis) for 895 *E. faecium* Group A1 isolates. Labelled genes are grouped by frequency along the x-axis which code biofilm-associated proteins (*bopD, clpP*); capsular polysaccharide biosynthesis (*cpsF*); fibrinogen (*fss3*) and general adhesion (*sgrA*); bile salt hydrolase (*bsh*) manganese transport (*psaA*); and collagen binding (acm*, scm, ecbA*).
